# Dramatic impacts on brain pathology, anxiety, and cognitive function in the knock-in APP^NL-G-F^ mouse model of Alzheimer disease following long-term voluntary exercise

**DOI:** 10.1186/s13195-022-01085-6

**Published:** 2022-09-30

**Authors:** Jogender Mehla, Scott H. Deibel, Hadil Karem, Shakhawat Hossain, Sean G. Lacoursiere, Robert J. Sutherland, Majid H. Mohajerani, Robert J. McDonald

**Affiliations:** 1 Canadian Centre for Behavioural Neuroscience, University of Lethbridge, 4401 University Dr W, Lethbridge, AB T1K 3M4 Canada; 2 Present address: Department of Neurological Surgery, Washington University School of Medicine, St. Louis, MO 63110 USA; 3 Present address: Department of Psychology, University of New Brunswick, POB 4400, Fredericton, NB E3B 3A1 Canada

**Keywords:** Alzheimer disease, APP^NL-G-F^ mice, Choline acetyltransferase, Cognitive dysfunction, Microgliosis, Physical exercise

## Abstract

**Background:**

An active lifestyle is associated with improved cognitive functions in aged people and may prevent or slow down the progression of various neurodegenerative diseases including Alzheimer’s disease (AD). To investigate these protective effects, male APP^NL-G-F^ mice were exposed to long-term voluntary exercise.

**Methods:**

Three-month-old AD mice were housed in a cage supplemented with a running wheel for 9 months for long-term exercise. At the age of 12 months, behavioral tests were completed for all groups. After completing behavioral testing, their brains were assessed for amyloid pathology, microgliosis, and cholinergic cells.

**Results:**

The results showed that APP^NL-G-F^ mice allowed to voluntarily exercise showed an improvement in cognitive functions. Furthermore, long-term exercise also improved anxiety in APP^NL-G-F^ mice as assessed by measuring thigmotaxis in the Morris water task. We also found reductions in amyloid load and microgliosis, and a preservation of cholinergic cells in the brain of APP^NL-G-F^ mice allowed to exercise in their home cages. These profound reductions in brain pathology associated with AD are likely responsible for the observed improvement of learning and memory functions following extensive and regular exercise.

**Conclusion:**

These findings suggest the potential of physical exercise to mitigate the cognitive deficits in AD.

## Background

Alzheimer disease (AD), the most common form of dementia, is a progressive neurodegenerative disease affecting elderly populations worldwide. AD is characterized by extra- and intra-cellular amyloid-beta (Aβ) plaques deposition and formation of neurofibrillary tangles (NFT) inside the neurons, synaptic loss, and severe cognitive decline [1–3]. Several non-genetic risk factors such as diabetes, obesity, hypertension, brain injuries, depression, or physical inactivity are associated with increased risk of AD worldwide [4–7]. Available pharmacological therapies provide only brief symptomatic relief [8]. Several epidemiological and clinical studies revealed that education, occupation, and physical activity can improve cognitive ability in healthy older people and provide protection against the development and progression of AD [6, 9–13].

Several epidemiological studies have already reported that physical activity significantly reduced the risk of dementia [14–16]. For example, people involved in mentally challenging activity and physical exercise during young and middle adulthood have four times less chance of getting AD compared with control subjects not engaging in such activities [11]. Additionally, people participating in leisure-time physical activity during middle age for at least twice a week showed reduced risk of dementia and AD, when compared with people not exercising at this age [17, 18]. Furthermore, several experimental studies have reported that physical activity prevents AD progression and improved cognitive functions by reducing Aβ pathology and amyloid angiopathy [19–23].

Mice exposed to social, physical, and cognitive training show a protective effect against cognitive impairment, decreased brain Aβ burden, and enhanced hippocampal synaptic immunoreactivity [24, 25]. Additionally, physical exercise in isolation has been shown to reduce AD pathology and improve memory in various murine models of AD [26, 27].

These and other animal studies show the potential benefits of physical exercise in reducing AD pathology and associated cognitive impairments. However, there are some caveats associated with this work including issues surrounding how accurately the mouse models of AD used in these studies mimic the brain changes in human AD. A second issue is the limited range of AD pathology assessed in these experiments.

The first issue, as noted, is that most mouse models of AD have overexpressed amyloid precursor protein (APP), or APP and presenilin1 (PS1) which leads to the accumulation of unusual fragments generated by α-secretase, such as C-terminal fragment-β (CTF-β). CTF-β is more toxic than Aβ and CTF-β does not accumulate in human AD brains. A recent study estimates that the neuropathological features of these mouse models are due to artifacts related to APP overexpression [28] and may explain the lack of translational success in clinical trials. We have been using a second-generation AD model recently developed at the Riken [28] which has a modified APP gene that has humanized Aβ sequence with three mutations in APP^NL-G-F^. This mouse model produces robust age-related spread of Aβ aggregates and cognitive problems with endogenous levels of APP. It is possible that the beneficial effects of exercise in traditional AD mouse models are due to reductions in these unusual protein fragments not seen in human AD.

A second issue we want to address in the present experiments is the focus of earlier studies on the effects of exercise on the two commonly studied pathologies associated with AD, Aβ plaques, and neurofibrillary tangles (NFT) [2, 3]. However, other brain pathologies are linked to the etiology of AD, including activated microglia and reactive astrocytes and other neuroinflammatory markers such as interleukin [29, 30]. Additionally, cholinergic cell death in nucleus basalis was also considered as one of the main markers of AD [31]. Previously, it has also been reported that AD patients have low levels of acetylcholine in the brain [32, 33]. Cholinergic dysfunction in the cortex and hippocampus regions is highly correlated with cognitive decline in AD [34]. Accordingly, we will employ a wider panel of AD pathology than most of the other studies in this area of research.

In the present study, we investigated the potential beneficial effects of long-term voluntary exercise on cognitive functions and brain pathology found in the APP^NL-G-F^ mouse model of AD. For the voluntary exercise experiment, individual mice (3 months old) were kept in a cage with a running wheel for 9 months. At 12 months of age, the subjects were tested on the Morris water task (MWT), novel object recognition (NOR), and a fear conditioning (FC) task to assess learning and memory functions mediated by brain networks centered on the hippocampus (HPC), perirhinal cortex (PRhC), and the amygdala (AMYG) respectively. After completion of behavioral tests, histology was completed to assess any changes in amyloid pathology, a microglial marker for neuroinflammation, and cholinergic cells.

## Materials and methods

### Animals and experimental design

**Fig. 1:**
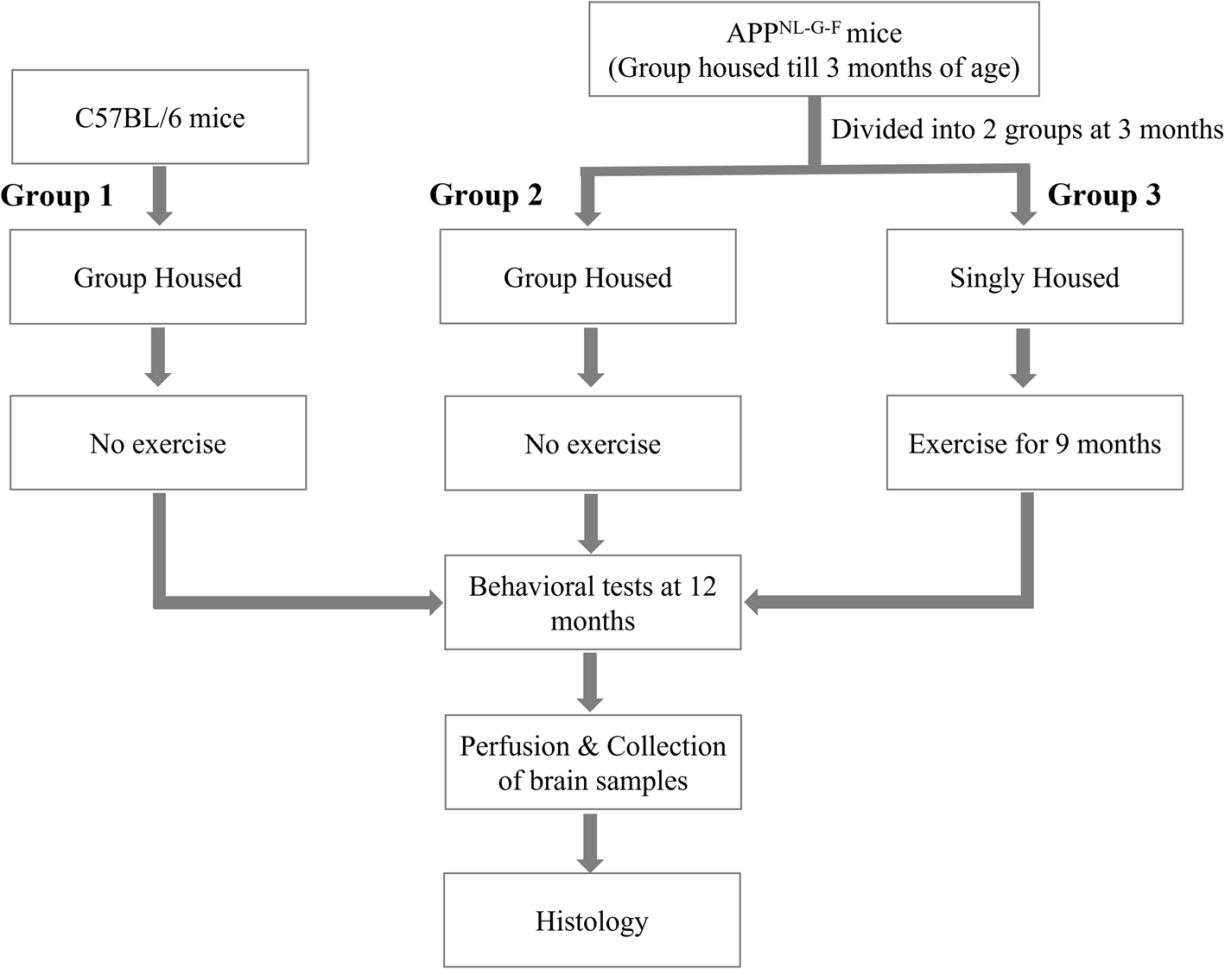
Flow chart for a detail experimental plan and groups for the current study

APP knock-in (APP-KI; APP^NL-G-F/NL-G-F^) mice were provided as a gift by the laboratory of Dr. Saido at the RIKEN Center for Brain Science, Japan. APP-KI mice were generated on a C57BL/6 background. A colony of these mice have been maintained at the vivarium at the Canadian Centre for Behavioral Neuroscience, University of Lethbridge. Mice were genotyped using ear notching method. In the present study, only homozygous APP^NL-G-F^ mice were used. In each cage, four mice were kept in a controlled environment with free access to food and water. At 3 months of age, 15 APP^NL-G-F^ mice were singly housed and were given access to a running wheel in their home cage for 9 months. As they were single housed, we checked each mouse for their health status by looking at their fur and eye condition. Age-matched littermates APP^NL-G-F^ (*n* = 10) and background controls (*n* = 10) were not singly housed to rule out the negative impact of social isolation on cognitive performance of these mice. Therefore, these mice were kept in group of 4 mice per cage and a running wheel was also not provided to their cages for the same 9 months. In total, we used 35 male mice that were 3 months old at the beginning of the study [group 1—10 C57BL/6 age matched normal control + no exercise; group 2—10 APP^NL-G-F^ + no exercise (APP-NE); group 3—15 APP^NL-G-F^ + exercise (APP-Ex)]. Age-matched non-littermate wild-type mice (C57BL/6) were used as a normal control. A detail experimental plan and groups are shown as flow chart (Fig. 1). When these mice reached 12 months of age, we performed various behavioral tests including MWT, NOR, and fear conditioning tests. After completing behavioral testing, we injected methoxy-XO4 (10 mg/kg) intraperitoneally to stain amyloid plaques in their brain [35, 36]. Twenty-four hours after this injection, mice were perfused, and the brains extracted for histology. We also performed immunostaining for microglial and choline acetyltransferase (ChAT) cells. The behavioral and histological analysis was performed by an experienced researcher blinded to the experimental groups.

#### Behavioral experiments

##### Morris water task (MWT)

**Fig. 2:**
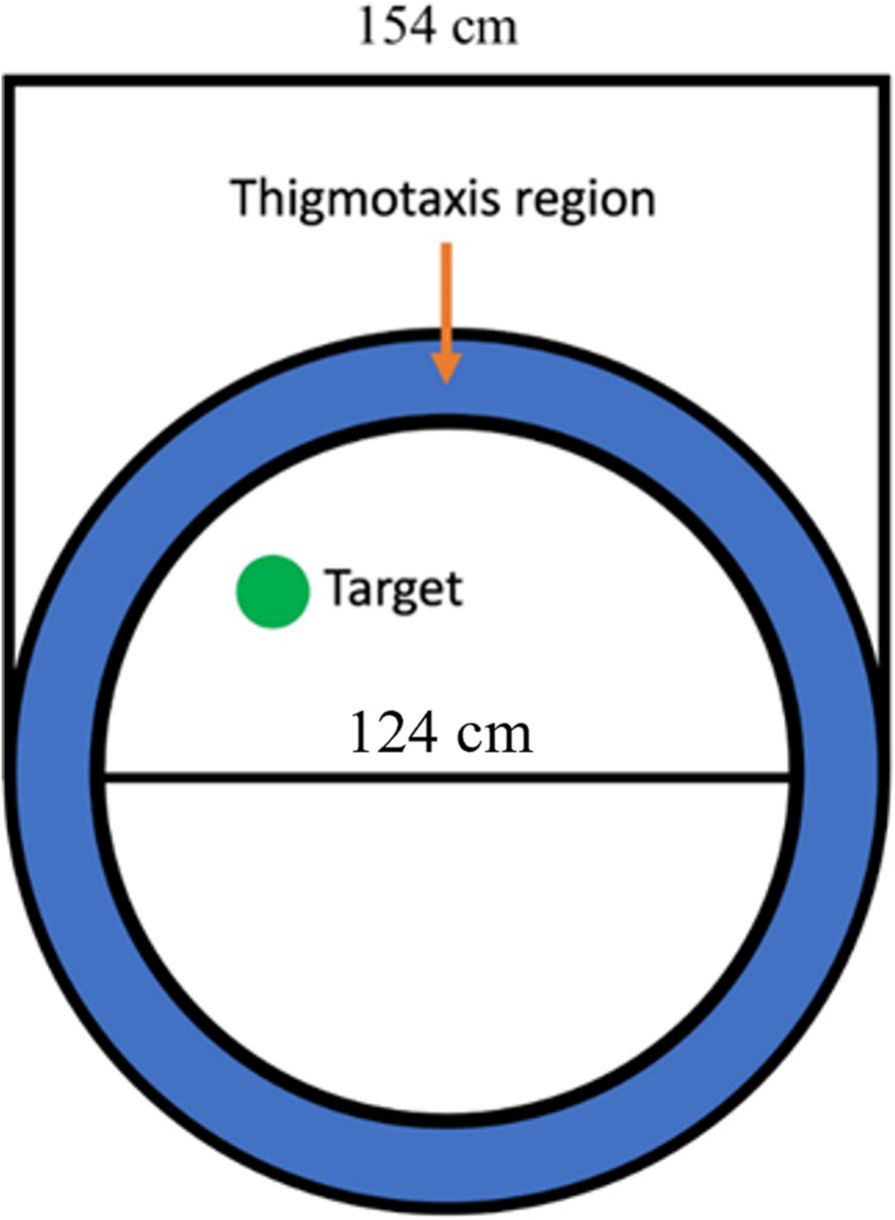
Representative pool schematic showing the size of the outer region of pool and the inner region. Any swimming in the thigmotaxis region (blue) was counted as time in thigmotaxis. Adopted from the ReadMe in the wtr2100 documentation

MWT experiments were conducted as reported previously [37, 38]. In brief, water maze experiments were performed in a circular tank (154 cm in diameter, 50 cm deep). The tank was filled to a depth of 40 cm with water and made opaque by adding non-toxic white paint and water temperature was maintained at 22±1°C. A circular platform (11 cm radius) was kept 0.5–1 cm below the water surface in a fixed location in the middle of one quadrant. The water tank was divided into four equal quadrants. Three distinct cues of different geometry were placed around the tank. On each acquisition day, mice received four distributed training trials from each quadrant for 8 days. The trial was completed once the mouse found the platform or 60 s had elapsed. If the mouse failed to find the platform on a given trial, the mouse was guided by the experimenter to the platform location. The latency to find the platform was used as an indicator of learning ability of the mice. Swim speed was analyzed to rule out the involvement of motor function as a confounding factor. Thigmotaxis behavior of experimental mice during acquisition phase was also analyzed. Briefly, the HVS data for swim pattern was collected and analyzed using wtr2100 to determine the thigmotactic time. The wrt2100 documentation states that thigmotactic swimming is defined as the amount of time when the swim path was restricted to a region around the pool that was proportional to the pool size. The ratio for this experiment was 0.8. The pool was 154 cm, meaning that if the mouse was swimming within the 124 to 154 cm region around the pool, this time was counted as the mouse being thigmotactic (Fig. 2). A single probe trial was performed on the ninth day to assess spatial memory performance. During the probe trial, mice were released from a start position located in the opposite quadrant to the quadrant in which the platform had been located during training. The data collected during the probe trial was analyzed measuring the time spent by mice in the target quadrant. Furthermore, average proximity was used to assess the spatial accuracy of the search strategy during the probe trial.

##### Novel object recognition (NOR) test

The test was performed as described previously [37, 38]. The advantage of using this test is that it does not require external motivation, reward, or punishment. It is based on the principle that mice will explore novel items. Specifically, when mice are familiarized with two similar objects during the training day, they will spend more time exploring a novel object on a subsequent test day, when in a familiar environment. This pattern of behavior clearly indicates that the mice formed representations of the objects during training and noticed the presence of a novel object during testing [39]. A white plastic square box (L × W × H; 52×51×30 cm) was used as the environment for the object recognition test. Briefly, mice were brought from their home cage to the experimental room and familiarized with the testing box for 5 min daily for 2–3 days. Training was conducted 24 h after the last acclimatization day. Two familiar objects were cleaned with 70% isopropyl alcohol to mask any previous odor cues and allowed to dry completely. A video camera was used to record the mice’s behavior for further analysis. The mice were put into the testing box for 10 min to explore both familiar objects. A test session was conducted 24 h later in which one of the familiar objects was replaced with a novel object, differing in geometry and texture. Mice were individually placed in the testing box for 5 min to explore the objects and their behavior was recorded. If the nose of the mouse was within 1–2 cm of the object, it was scored as in contact with an object. Time for a mouse moving over the top of the object, sitting on the top of the object, or looking past the object was not included during the data analysis. After each mouse, the testing box was cleaned and the objects were wiped with 70% isopropyl alcohol to mask odor cues. The data was analyzed by measuring the exploration time for the familiar and novel object during training and testing days. The investigation ratio was calculated using following formula:

$$\mathrm{Investigation}\;\mathrm{ratio}:\;\mathrm{For}\;\mathrm{Familiar}\;\mathrm{Object}-\;T_{\mathrm F}/(T_{\mathrm F}+T_{\mathrm N});\mathrm{For}\;\mathrm{Novel}\;\mathrm{Object}-\;T_{\mathrm N}/(T_{\mathrm N}+T_{\mathrm F}),\;\mathrm{where}\;T_{\mathrm N}\;\mathrm{is}\;\mathrm{time}\;\mathrm{to}\;\mathrm{explore}\;\mathrm{novel}\;\mathrm{object}\;\mathrm{and}\;T_{\mathrm F}\;\mathrm{is}\;\mathrm{time}\;\mathrm{to}\;\mathrm{explore}\;\mathrm{familiar}\;\mathrm{object}$$

##### Fear conditioning (FC) test

FC was conducted as described in previous studies [37, 38]. This test is used to assess the amygdala- and hippocampus-associated memory in rodents. FC was conducted in an acrylic square chamber. A video camera was used to record the mice’s behavior for further analysis. The floor of the chamber consisted of stainless-steel rods that were connected to a shock generator for the delivery of a foot-shock. A speaker was used to deliver the tone stimulus. Prior to conditioning, the chamber was cleaned with a 1% Virkon solution to mask any previous odor cues. On the conditioning day, mice were brought individually from their home cage into a testing room and allowed to sit undisturbed in their cage for 10 min. Mice were then kept in the conditioning square chamber and allowed to explore for 2 min before the onset of the tone (20 s, 2000 Hz). In the delay conditioning test, a shock (2 s, 0.5 mA) was given in the last 2 s of tone duration. Mice received five delayed conditioning trials, with a 120-s intertrial interval (ITI). The mice were taken from the conditioning chamber 1 min after the last shock and returned to their home cages. After 24 h, the tone test was conducted in a triangular chamber located in different room that was geometrically different from the conditioning chamber to assess conditioning to the tone in the absence of the training context. The triangular chamber was cleaned with a 70% isopropyl solution before each mouse was tested. For the tone test, three 20-s tones were given after a 2-min baseline period. Each tone presentation was separated by a 120-s ITI. The mice were taken from the triangular chamber 1 min after the last tone presentation and returned to their home cages. The freezing response was measured using a time sampling procedure in which an observer scored the presence or absence of the freezing response for each mouse at every 2-s interval. Twenty-four hours after the tone test, a context test was conducted by placing each mouse back in the original conditioning box for 5 min. During this test, freezing was scored for each mouse at every 5-s interval. Data was transformed into a percent freezing score by dividing the number of freezing observations by the total number of observations and multiplying by 100.

### Histology

Upon completion of behavioral tests, mice were injected with methoxy-XO4 (10 mg/kg, i.p) as described in a previous study [35, 36]. Twenty-four hours after the injection, mice were transcardially perfused with phosphate-buffered saline (PBS) followed by 4% paraformaldehyde (PFA). Brains were extracted and post-fixed for 24 h in 4% PFA at 4 °C. The brains were rinsed with PBS and then transferred to a 30% sucrose solution. For histology, 4–5 mice were randomly selected and three sections for each brain region were analyzed and the data was then averaged. We and others have used these subject numbers and obtained more than sufficient power for statistical analysis [37].

#### Quantification of amyloid plaques in mice brain

We assessed amyloid pathology in different brain regions such as medial prefrontal cortex (mPFC), hippocampus (HPC), retrosplenial area (RSA), perirhinal cortex (PRhC), and cortical amygdalar area (CAA). Therefore, we used brain atlas section +1.94mm for mPFC and atlas section −3.08mm for HPC, RSA, PRhC, and CAA [40]. Fixed brains were coronally sectioned at 40μm using a freezing microtome. Brain sections were mounted on a slide and air dried for 15–20 min. Later, sections on the slides were washed twice with tris-buffered saline (TBS) for 5 min each. Then, sections on the slides were coverslipped with Vectashield H-1000 (Vector Laboratory). Finally, whole slides were imaged using a NanoZoomer microscope with 20× objective magnification (NanoZoomer 2.0-RS, HAMAMTSU, JAPAN). The images were analyzed using ImageJ (US National Institutes of Health, Bethesda, MD) and ilastik software [41]. This software automatically gives the plaque number and size corresponding with each discrete plaque. The plaque number was quantified according to the plaque size (less than or more than 4 μm). In line with Hefendehl and colleagues, 87% of newly generated plaques are small, having a radius of < 4 μm [42]. ImageJ (US National Institutes of Health, Bethesda, MD) was used to determine the plaque area.

#### Immunostaining for microglial and choline acetyl transferase (ChAT)

Brains were serially sectioned coronally at 40 μm on a freezing microtome. Immunohistochemical procedures for IBA-1 and ChAT were performed as previously described [28, 37]. In brief, sections were fixed on positively subbed slides. Brain sections on the slides were washed in TBS and then blocked for 2 h in TBS containing 0.3% Triton-X and 3% goat serum. The sections were incubated for 24 h in primary antibody (prepared in TBS with 0.3% Triton-X) at room temperature in a dark humid chamber on the shaker. The following primary antibodies were used: rabbit anti-ChAT (monoclonal, Abcam, ab178850, 1:5000); anti-IBA-1 (Rabbit, SAF4318, 019-19741, Wako). Following incubation, three 10-min washes were done, and sections were incubated with secondary antibodies for 24 h. The following secondary antibodies were used: goat anti-rabbit-alexa-594 (IgG (H+L), A11037, Invitrogen, 1:1000 for ChAT and IBA-1). Following incubation with secondary antibodies, three 10-min washes were given. Finally, the ChAT sections were incubated with DAPI (1:2000 of the 20ug/ml stock in TBS) for 45–60 min. After incubation, a single 5-min wash was given. Then, sections on the slides were coverslipped with Vectashield H-1000. Later, the slides were sealed with nail polish. Finally, whole slides were imaged using the NanoZoomer 2.0-RS microscope with 20× objective magnification. The images were analyzed using ImageJ and ilastik software [41].

### Statistical analysis

The statistical analysis was done using SPSS statistical software package, version 22.0. Results are given as mean ±SEM. A mixed model ANOVA with day as the repeated measures factor and group as the between subjects’ factor was used to analyze water task acquisition. One-way ANOVA with Bonferroni post hoc test was used to find significant differences between the experimental groups for object recognition, fear conditioning, microgliosis and ChAT markers. Additionally, a paired *t*-test was used to find statistically significant differences between familiar and novel objects in the object recognition test. Independent *t* test was used for the comparison of amyloid pathology between experimental groups. A *p* value < 0.05 was considered as statistically significant.

## Results

### Effect of long-term voluntary exercise on learning and memory functions of APP^NL-G-F^ mice in MWT

**Fig. 3:**
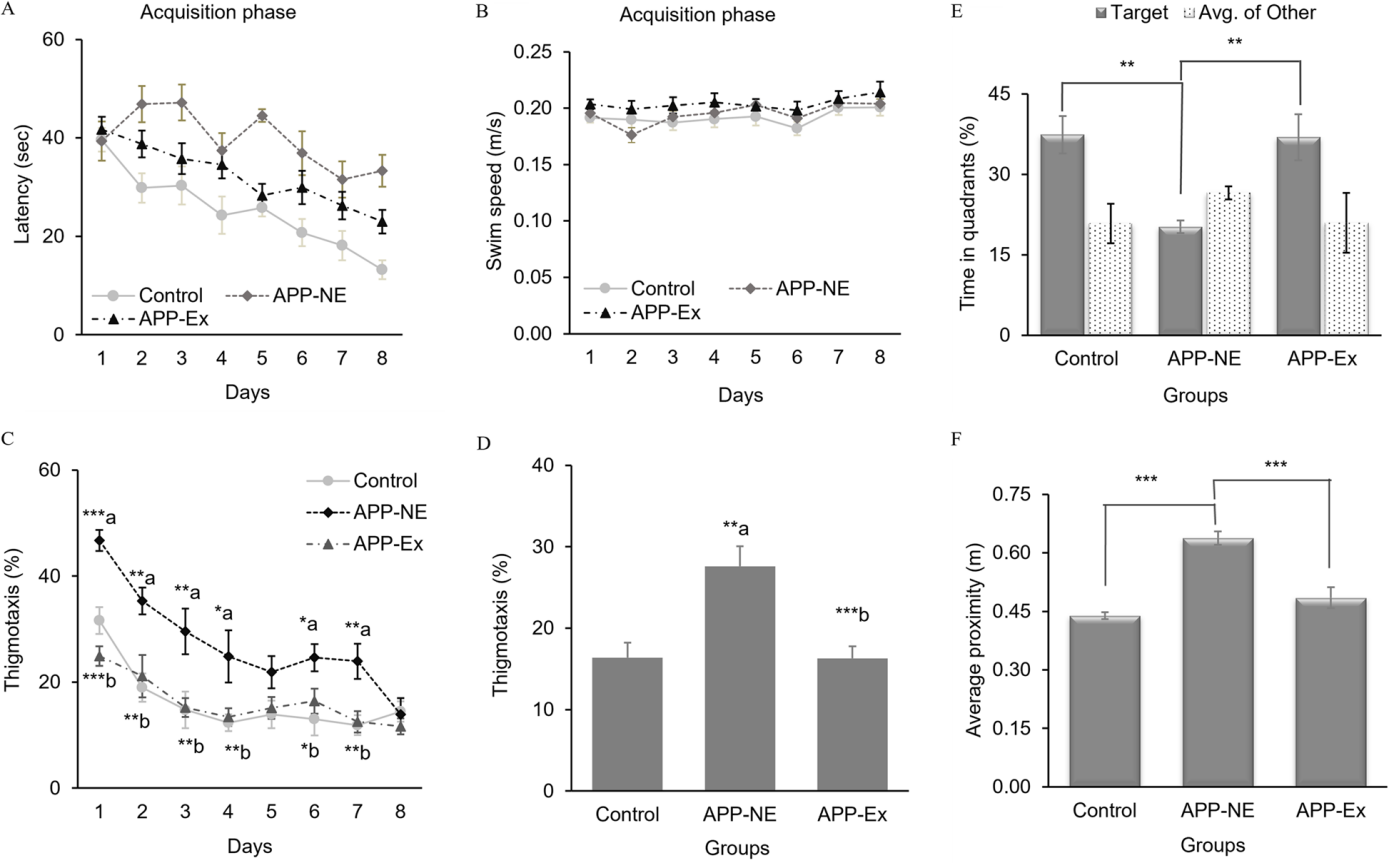
Effects of long-term voluntary exercise on spatial learning and memory functions of 12-month-old APP^NL-G-F^ mice in the MWT. **A** Mean latency to find the hidden escape platform during the acquisition phase. **B** Mean swim speed during the acquisition phase. **C** Percent thigmotaxis shown by mice on each day during the acquisition phase. **D** Percent thigmotaxis shown by mice during the acquisition phase (average of days 1–8). **E** Percent time spent by mice in target quadrant and average of other quadrants during the probe trial. **F** Average proximity to platform during probe trial. Data is presented as mean±SEM. *p < 0.05, **p < 0.01, ***p < 0.001 are considered as statistically significant. a, as compared to control group; b, as compared to APP-NE group. Control group, C57BL/6 (*n*= 10). APP-NE group, APP^NL-G-F^ with no exercise (*n* = 10). APP-Ex group- APP^NL-G-F^ mice exposed to exercise (*n* = 15)

A mixed model ANOVA with day as the repeated measures factor and group as the between subjects’ factor was used to analyze MWT acquisition. Latencies significantly decreased across training days (Fig. 3A), suggesting learning occurred (*F* (7,224) = 12.262, *p* < 0.001). Performance differed between the groups (*F* (2,32) = 23.586, *p* < 0.001), but there was not a significant group × day interaction. Bonferroni multiple comparisons conducted to parse out the main effect indicated that controls performed significantly better than the APP-NE (*p* = 0.001) and APP-Ex groups (*p* = 0.001; Fig. 3A). No significant difference in the swim speed of experimental groups across acquisition training days was found (Fig. 3B).

In the probe trial, there were main effects of quadrant (*F* (1,32) = 6.341, *p* = 0.005) and group (*F* (1,32) = 9.070, *p* = 0.005) and a quadrant × group interaction (*F* (1,32) = 6.369, *p* = 0.005). Follow-up Bonferroni post hoc tests found that the controls spent more time in the target quadrant than APP-NE (*p* = 0.001), but not APP-Ex animals (Fig. 3E). APP-Ex animals also spent more time in the target quadrant than the APP-NE animals (*p* = 0.001; Fig. 3E). Similarly, the proximity measure also differed between the groups (*F* (2,32) = 18.950, *p* < 0.001), with both the controls (*p* < 0.001) and APP-Ex (*p* < 0.001) groups having a closer platform proximity than the APP-NE animals (Fig. 3F).

Overall, these results suggest that exercise reversed the functional impairments in the APP^NL-G-F^ mice on the spatial version of the MWT, a task repeatedly shown to be sensitive to hippocampal dysfunction. The lack of a statistically significant effect during acquisition but large effects of the probe trial and spatial specificity is consistent with work done by Bannerman and colleagues [43] in which they showed larger impacts of HPC lesions on the probe trial compared to training trials.

An analysis of thigmotaxia was also assessed. Thigmotaxia is the tendency of rodents to avoid venturing away from the pool wall. This behavior can emerge because of impaired learning and memory abilities [44], anxiety [45], sex hormones [46], and altered executive functions leading to perseveration [47]. In the present study, we found that training during acquisition phase caused significant reduction in thigmotaxis behavior of all experimental groups (*F* (7,224) = 12.262, *p* < 0.000; *η*^2^ = 0.494). APP-NE group showed more thigmotaxia than control group (Fig. 3C). Bonferroni test analysis revealed no significant difference in thigmotaxia between all experimental groups on day 8 of acquisition phase (Fig. 3C). APP-Ex group showed a significant reduction [(*F*_(2,32)_ = 27.495, *p* = 0.000 for day 1); (*F*_(2,32)_ = 7.077, p = 0.003 for day 2); (*F*_(2,32)_ = 7.117, p = 0.003 for day 3); (*F*_(2,32)_ = 5.448, *p* = 0.009 for day 4); (*F*_(2,32)_ = 2.668, *p* = 0.085 for day 5); (*F*_(2,32)_ = 4.484, *p* = 0.019 for day 6); (*F*_(2,32)_ = 7.155, *p* = 0.003 for day 7); (*F*_(2,32)_ = 0.542, *p* = 0.587 for day 8)] in thigmotaxia compared to the APP-NE group indicating that exercise improved the thigmotaxis behavior of APP^NL-G-F^ mice (Fig. 3C). No significant difference in thigmotaxia of control and APP-Ex group was found during acquisition phase. Additionally, we also analyzed average thigmotaxis behavior of experimental groups across the acquisition phase (days 1–8) and a statistically significant difference among experimental groups (*F*_(2,32)_ = 11.113, *p* = 0.000; *η*^2^ = 0.410) was found (Fig. 3D). Mice in the APP-NE group showed significantly (*p* < 0.01) more thigmotaxia as compared to control mice (Fig. 3D). However, APP^NL-G-F^ mice exposed to physical exercise in the APP-Ex group showed significant (*p* < 0.001) reduction in thigmotaxia as compared to the APP-NE group (Fig. 3D).

Taken together, the pattern of effects and statistical analysis show that voluntary exercise dramatically improved spatial learning and memory functions rendered dysfunctional in APP^NL-G-F^ mice. These effects were particularly pronounced on the spatial probe trial and on spatial specificity measures. These results suggest that exercise improves hippocampal functions specifically as acquisition and retention of the spatial version of the water task has been consistently shown to be mediated by a network centered on the hippocampus [48, 49].

### Effect of long-term voluntary exercise on the memory function of APP^NL-G-F^ mice in a novel object recognition test

**Fig. 4:**
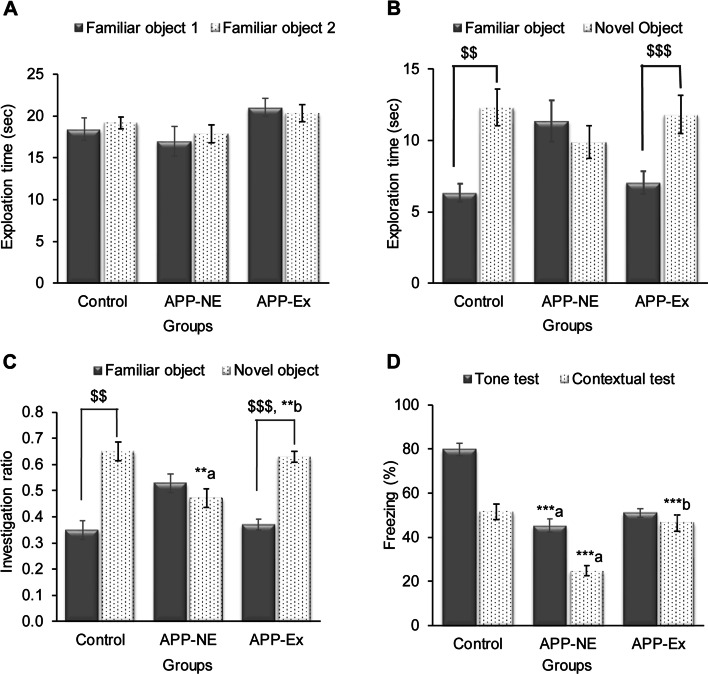
Effect of long-term voluntary exercise on learning and memory functions of 12-month-old APP^NL-G-F^ mice in the novel object recognition (NOR) and fear conditioning tests. **A** Exploration time on the training day. **B** Exploration time on the testing day. **C** Investigation ratio for familiar and novel object. **D** Percent freezing for tone and contextual fear test. Data is presented as mean±SEM. **p < 0.01, ***p < 0.001; a, as compared with the control group. b, as compared with the APP-NE group. $$*p* < 0.01, $$$*p* < 0.001 as compared to familiar object. Control group, C57BL/6 (*n* = 10). APP-NE group, APP^NL-G-F^ with no exercise (*n* = 10). APP-Ex group, APP^NL-G-F^ mice exposed to exercise (*n* = 15)

In the object recognition test, control mice showed normal associated memory function whereas APP-NE mice did not (Fig. 4B, C). During the training, no significant difference was found in exploration time for object 1 and 2 in any experimental groups (Fig. 4A). However, on testing day, control mice explored a novel object for significantly (*p* < 0.01) longer time when compared to the familiar object (Fig. 4B). Furthermore, control mice showed a significantly (*p* < 0.01) higher investigation ratio for the novel object in comparison to the familiar object indicating these mice are spending more time exploring the novel object while the APP group did not, suggesting that the latter group had an object memory impairment (Fig. 4C). One-way ANOVA revealed statistically significant (*F*_(2,32)_ = 9.570, *p* = 0.001; *η*^2^ = 0.374) difference in investigation ratio among experimental groups. Bonferroni post hoc test indicated that the investigation ratio for the novel object was significantly (*p* < 0.01) lower in the APP-NE group in comparison with control (Fig. 4C). In contrast, APP^NL-G-F^ mice with physical exercise showed significantly (*p* < 0.001) higher investigation ratio for the novel object when compared with a familiar object which is also evidenced by significantly more exploration time for a novel object than familiar (Fig. 4B, C). Furthermore, the APP-Ex group also showed significantly (*p* < 0.01) higher investigation ratio for the novel object as compared to the APP-NE group (Fig. 4C).

### Effect of long-term voluntary exercise on memory function of APP^NL-G-F^ mice in fear conditioning

Figure 4D shows the results of the fear conditioning test. Control mice showed conditioned fear to an auditory cue and context associated with a fearful stimulus (foot-shock) while the APP mice did not (Fig. 4D). One way ANOVA indicated a statistically significant difference in freezing percent in tone (*F*_(2,32)_ = 46.992, *p* < 0.001, *η*^2^ = 0.597) and context (*F*_(2,32)_ = 14.537, *p* < 0.001, *η*^2^ = 0.476) conditioning tests among experimental groups. Consistent with these findings, Bonferroni post hoc analysis revealed that APP-NE mice showed significantly (*p* < 0.001) less freezing percentage to the tone and context tests when compared with the control mice (Fig. 4D). APP-Ex mice showed an improvement in memory function indicated by significant increases in percentage of freezing to the conditioned context (*p* < 0.001) in comparison with the APP-NE group (Fig. 4D). However, no improvement in memory function of the APP-Ex group was found in the auditory cue test (Fig. 4D).

Taken together, the functional assessment of long-term impacts of voluntary exercise on the APP^NL-G-F^ mouse model of AD suggests that the neural networks mediating spatial, object and fear memories are compromised in the APP^NL-G-F^ mouse and that repeated long-term voluntary exercise preserve cognitive domains associated with these brain areas.

### Effect of long-term voluntary exercise on amyloid pathology of APP^NL-G-F^ mice

**Fig. 5:**
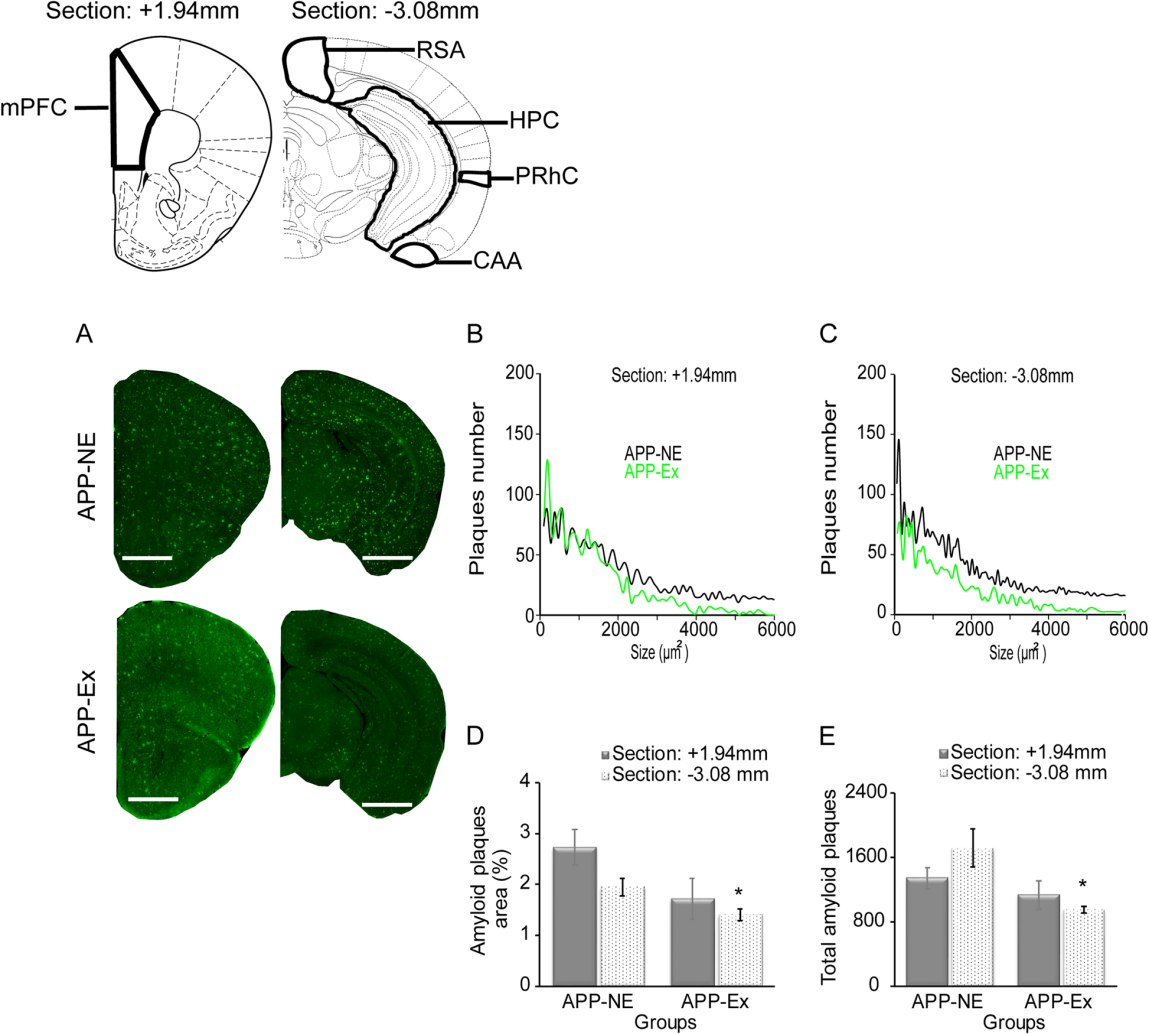
Amyloid plaque distribution in brain of 12-month-old APP^NL-G-F^ mice. **A** Photomicrographs of amyloid plaques stained with methoxy-XO_4_ (green) in the brain. **B**, **C** Corresponding distributions of plaque size by total number of plaques. **D** Percent amyloid plaque area in brain. **E** Total amyloid plaques number in the brain. Scale bars represent 1 mm for atlas section +1.94 mm and 2.5 mm for atlas section −3.08 mm. Data is presented as mean ± SEM. *p < 0.05—as compared to the APP-NE group. APP-NE group, APP^NL-G-F^ with no exercise (*n* = 4). APP-Ex group, APP^NL-G-F^ mice exposed to exercise (*n* = 4)

We measured amyloid pathology in different brain regions such as mPFC, HPC, RSA, PRhC, and CAA. Therefore, we used brain atlas section +1.94mm for mPFC and atlas section −3.08mm for HPC, RSA, PRhC, and CAA. Figure 5B and C show a representative distribution of Aβ plaques size by total number of plaques in brain sections of APP-NE (*n* = 4) and APP-Ex (*n* = 4) groups. Lowest distribution of Aβ plaques size by total number of plaques was observed in APP-Ex mice. Independent *t*-test showed statistically significant difference in amyloid percent area (*t*_(6)_ = 2.663, *p* < 0.05), and amyloid plaques number (*t*_(6)_ = 3.172, *p* < 0.05) in posterior brain atlas section −3.08mm among experimental groups. Additionally, exercise significantly (*p* < 0.05) decreased amyloid load in the brain of APP^NL-G-F^ mice (Fig. 5D, E).

**Fig. 6:**
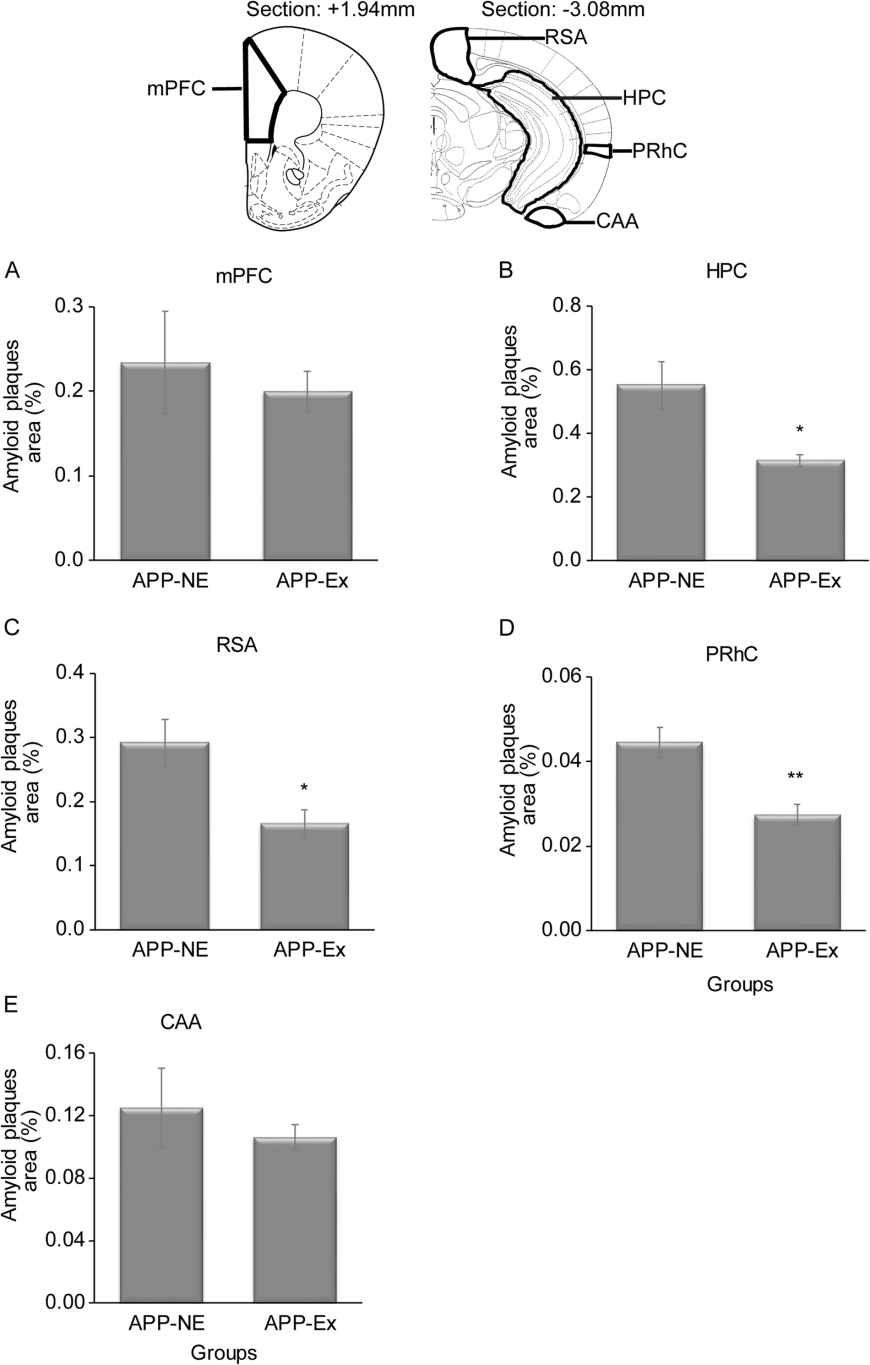
Amyloid plaque distribution in different brain regions of 12-month-old APP^NL-G-F^ mice. **A** Amyloid plaque area in medial prefrontal cortex (mPFC). **B** Amyloid plaque area in hippocampus (HPC). **C** Amyloid plaque area in retrospenial area (RSA). **D** Amyloid plaque area in perirhinal cortex (PRhC). **E** Amyloid plaque area in cortical amygdalar area (CAA). Data is presented as mean±SEM. *p < 0.05, **p < 0.01, as compared to the APP-NE group. APP-NE group, APP^NL-G-F^ with no exercise (*n* = 4). APP-Ex group, APP^NL-G-F^ mice exposed to exercise (*n* = 4)

We also investigated the deposition of Aβ plaques in various brain regions such as mPFC, HPC, RSCA, PRhC, and CAA as these brain regions are involved in various cognitive functions including learning and memory (Fig. 6A–E). No significant difference was found in amyloid load in mPFC (Fig. 6A) and CAA (Fig. 6E) brain areas of the APP-Ex group when compared with the APP-NE group. Independent *t*-test analysis indicated that APP-Ex mice showed significant decrease in Aβ plaques burden in HPC (*t*_(6)_ = 3.080, *p* < 0.05, Fig. 6B), RSA (*t*_(6)_ = 2.938, *p* < 0.05, Fig. 6C), and PRhC (*t*_(6)_ = 3.987, *p* < 0.01, Fig. 6D) when compared to the APP-NE group.

Additionally, we investigated amyloid burden in medial septum-diagonal band complex (MSDB), mainly responsible for cholinergic inputs to various brain regions especially HPC. Figure 9D displays a representative distribution of Aβ plaques size by total number of plaques in the MSDB complex of APP-NE and APP-Ex groups. APP-Ex mice showed the lowest distribution of Aβ plaques size by total number of plaques. Independent t-test analysis indicated that APP-Ex group showed significant reductions in Aβ plaques number (*t*_(6)_ = 11.552, *p* < 0.001, *η*^2^ = 0.957), and percent Aβ plaques area (*t*_(6)_ = 3.250, *p* < 0.05) when compared to APP-NE group (Fig. 9E, F).

The results of long-term voluntary exercise on amyloid pathology in the APP^NL-G-F^ mouse were clear. Specifically, amyloid pathology was drastically reduced in brain areas implicated in the learning and memory functions assayed in these same subjects, namely the HPC, RSC, PRhC, and MSDB regions.

### Effect of long-term voluntary exercise on brain microgliosis of APP^NL-G-F^ mice

**Fig. 7:**
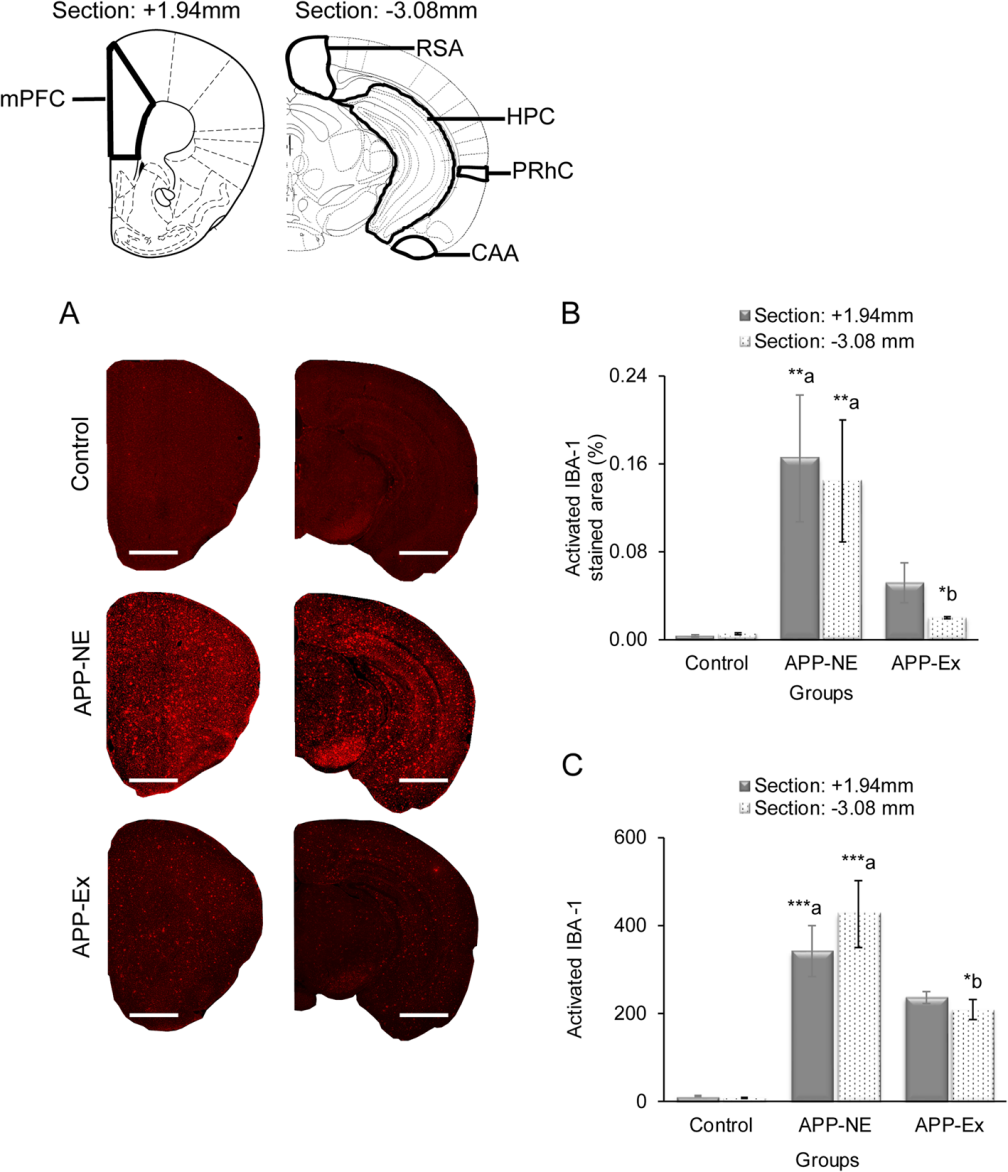
Microgliosis in brain of 12 months old mice. **A** Photomicrographs of activated IBA-1, a marker of microgliosis in brain. **B** Activated IBA-1 immunostained area in brain atlas section +1.94 mm and atlas section −3.08 mm. **C** Activated IBA-1 number in brain atlas sections +1.94 mm and atlas section -3.08 mm. Scale bars represent 1 mm for atlas section +1.94 mm and 2.5 mm for atlas section −3.08 mm. Data is presented as mean ± SEM. *p < 0.05, **p < 0.01, ***p<0.001; a, as compared to the control group; b, as compared to the APP-NE group. Control group, C57BL/6 (*n* = 5). APP-NE group, APP^NL-G-F^ with no exercise (*n* = 4). APP-Ex group, APP^NL-G-F^ mice exposed to exercise (*n* = 4)

Figure 7 shows increased microgliosis in the brain of mice in the APP-NE group when compared with the control group. Overall, physical exercise decreased microgliosis in the brains of the APP-Ex mice in comparison to that in the brains of APP-NE mice (Fig. 7B, C). One-way ANOVA indicated statistically significant differences in IBA-1 percent area [atlas section +1.94mm; (*F*_(2,10)_ = 6.721, *p* = 0.014, *η*^2^ = 0.856), and atlas section −3.08mm; (*F*_(2,10)_ = 6.675, *p* = 0.014, *η*^2^ = 0.836)] and number of activated IBA-1 [(atlas section +1.94mm; (*F*_(2,10)_ = 29.625, *p* < 0.001, *η*^2^ = 0.573), and atlas section −3.08mm; (*F*_(2,10)_ = 25.447, *p* < 0.001, *η*^2^ = 0.572)] among experimental groups. We also found significant increases in microgliosis in brain atlas section +1.94mm and atlas section −3.08mm of the APP-NE group compared to controls (Fig. 7B, C). Bonferroni post hoc analysis revealed that APP-Ex mice showed a significant decrease in microgliosis in comparison with APP-NE mice (Fig. 7B, C).

**Fig. 8:**
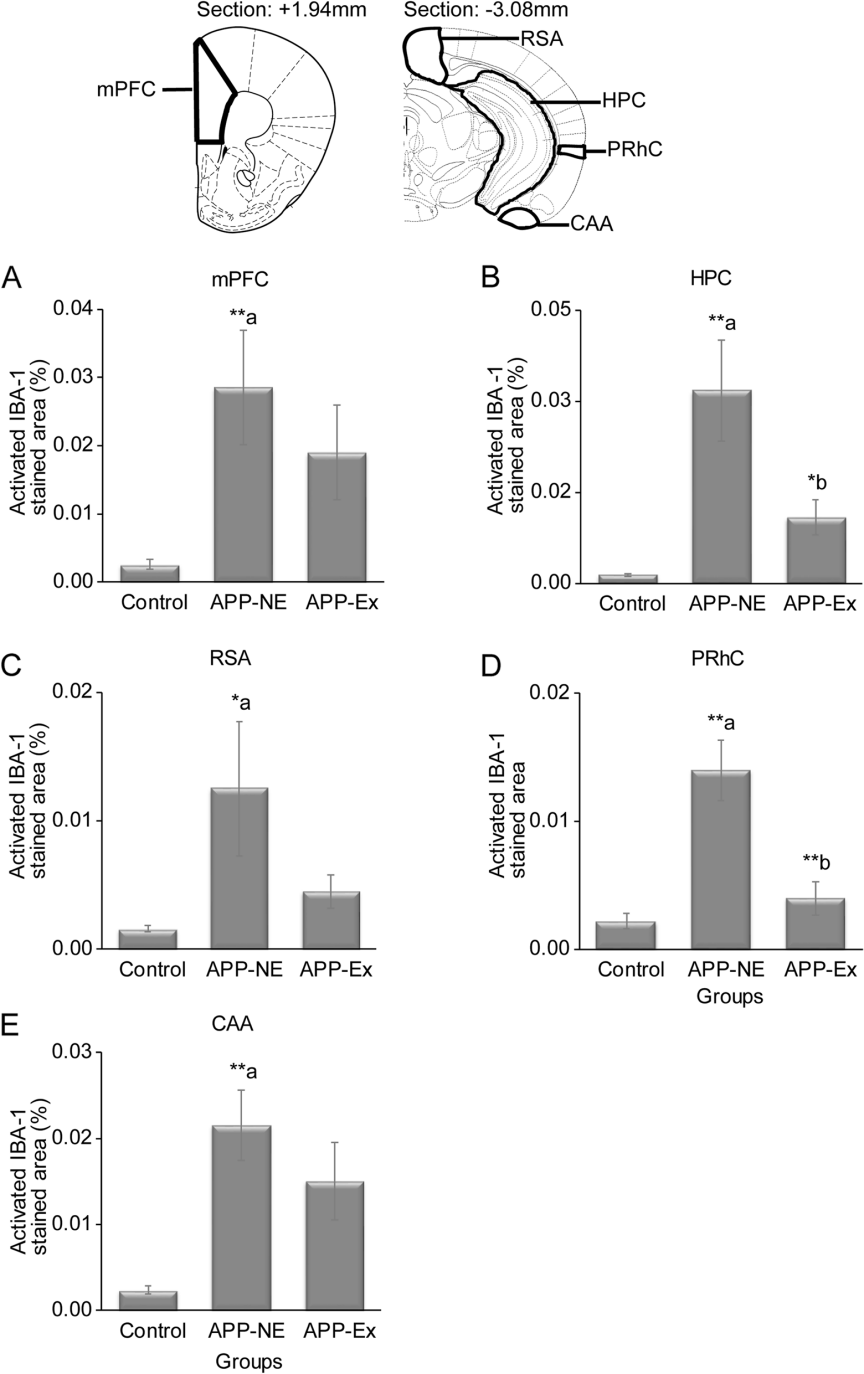
Microgliosis in different brain regions of 12-month-old mice. **A** Activated IBA-1 immunostained area in medial prefrontal cortex (mPFC). **B** Activated IBA-1 immunostained area in hippocampus (HPC). **C** Activated IBA-1 immunostained area in retrospenial area (RSA). **D** Activated IBA-1 immunostained area in perirhinal cortex (PRhC). **E** Activated IBA-1 immunostained area in cortical amygdalar area (CAA). Data is presented as mean ± SEM. *p < 0.05, **p < 0.01; a, as compared to the control group; b, as compared to the APP-NE group. Control group, C57BL/6 (*n* = 5). APP-NE group, APP^NL-G-F^ with no exercise (*n*= 4). APP-Ex group, APP^NL-G-F^ mice exposed to Exercise (*n* = 4)

We also assessed the microgliosis in different brain regions, which are involved in various cognitive functions and have been shown to be compromised in humans with AD. One-way ANOVA showed statistical significant difference in IBA-1 percent area in mPFC (*F*_(2,10)_ = 5.156, *p* = 0.029); HPC (*F*_(2,10)_ = 11.203, *p* = 0.003, *η*^2^ = 0.691); RSA (*F*_(2,10)_ = 3.953, *p* = 0.054); PRhC (*F*_(2,10)_ = 18.053, *p* < 0.001, *η*^2^ = 0.783); and CAA (*F*_(2,10)_ = 9.440, *p* = 0.005, *η*^2^ = 0.654) of experimental groups. IBA-1 percent area was significantly higher in the APP-NE group in comparison with control mice (Fig. 8A–E). After Bonferroni post hoc analysis, a significant reduction in microgliosis was also observed in HPC (*p* < 0.05), and PRhC (*p* < 0.01) brain regions of APP-Ex group when compared to the APP-NE (Fig. 8B, D). We also observed a reduction in microgliosis in other brain areas of APP-Ex mice but the difference was not statistically significant when compared with APP-NE.

**Fig. 9:**
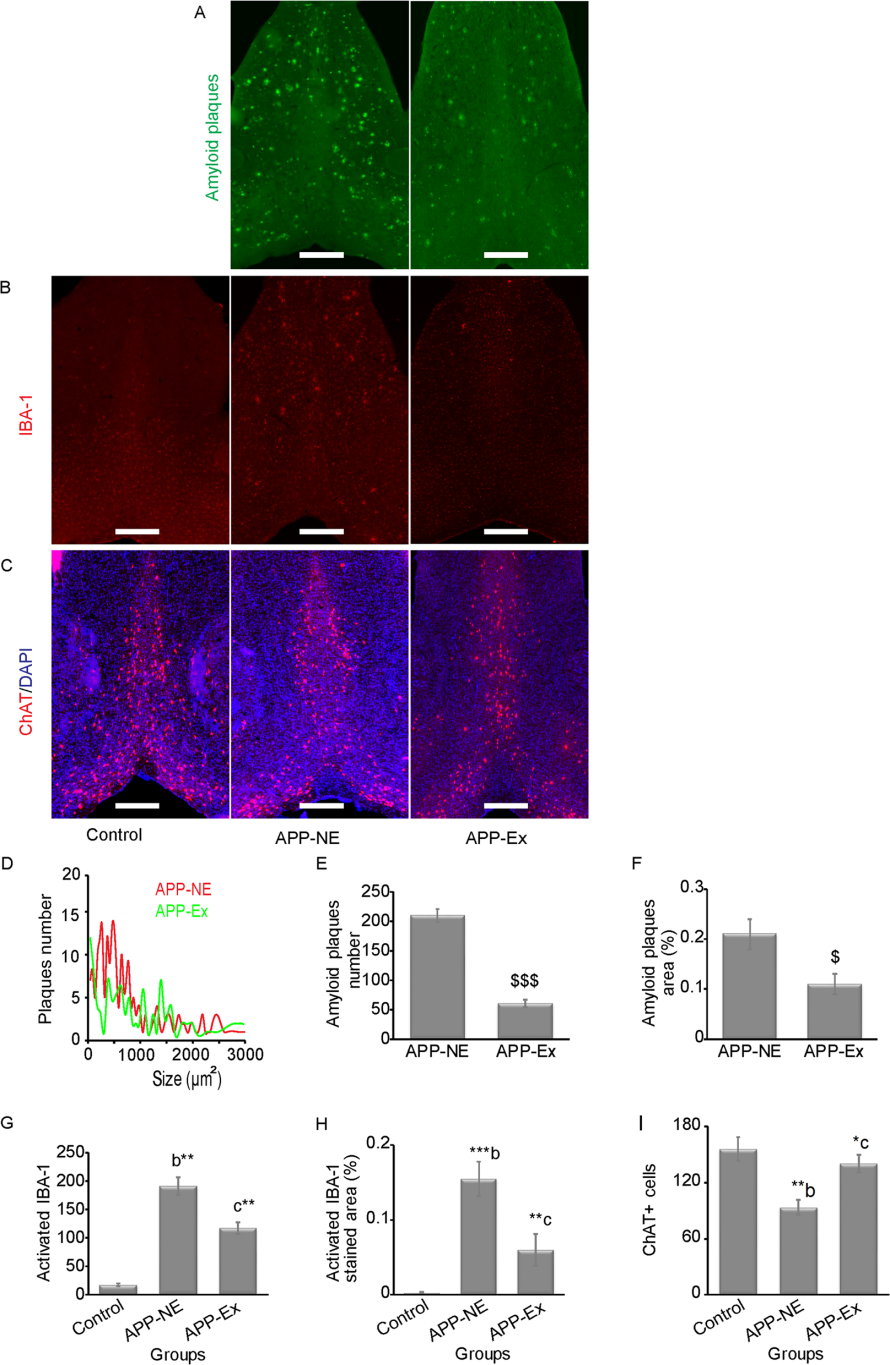
Amyloid pathology, microgliosis, and ChAT^+^ cells in the medial septum-diagonal band (MSDB) complex of the basal forebrain of 12-month-old mice. **A** Photomicrographs of amyloid plaques stained with methoxy-XO_4_ (green) in MSDB complex. **B** Photomicrographs of immunostaining of activated IBA-1 (red) in MSDB complex. **C** Photomicrographs of immunohistochemistry staining of choline acetyl transferase (ChAT) in MSDB complex. ChAT, a cholinergic marker stained with monoclonal rabbit anti-ChAT antibody (red); DAPI, stained the nuclei (blue). Scale bar, 500 μm for whole sections. **D** Corresponding distributions of plaque size by total number of plaques. **E** Total amyloid plaques number in MSDB complex. **F** Percent amyloid plaque area in MSDB complex. **G** Activated IBA-1 number in MSDB complex. **H** Activated IBA-1 percent area in MSDB complex. **I** Quantification of ChAT in MSDB complex. Data is presented as mean±SEM. *p < 0.05, **p < 0.01, ***p < 0.001 are considered as statistically significant. $, as compared to APP-NE group; b, as compared to control group; c, as compared to APP-NE group. Control group, C57BL/6 (*n* = 5). APP-NE group, APP^NL-G-F^ with no exercise (*n* = 4). APP-Ex group, APP^NL-G-F^ mice exposed to exercise (*n* = 4)

We also assessed microgliosis in the MSDB complex. One-way ANOVA indicated statistically significant difference in number of activated IBA-1 (*F*_(2,10)_ = 21.173, *p* < 0.001, *η*^2^ = 0.809) and IBA-1 percent area (*F*_(2,10)_ = 22.280, *p* < 0.000, *η*^2^ = 0.817) in MSDB of experimental groups. APP-NE mice showed increases in microgliosis in the MSDB complex evidenced by increased activation of IBA-1 and IBA-1 percent area when compared to the control group (Fig. 9G, H). Bonferroni post hoc analysis revealed that the APP-Ex group showed significant reduction in microgliosis (*p* < 0.01) in the MSDB complex in comparison with the APP-NE group (Fig. 9G, H).

In summary, APP^NL-G-F^ mice showed elevated microgliosis in the same brain regions that are functionally compromised in this AD mouse model including HPC and PRhC as revealed via behavioral analysis including spatial memory in the water task and NOR. HPC is considered a central structure in a complex network important for spatial learning and memory and the PRhC is considered a central structure in a neural network crucial for object recognition [50]. The most convincing evidence for the idea that the HPC and PRhC are key components of neural networks involved in spatial navigation and visual recognition memory respectively is various double dissociations of neurotoxic lesions and reversible inactivations on these and other tasks [51–53]. Further, the results showed that long-term voluntary exercise reduces elevated microgliosis in these same regions and these subjects’ spatial memory and NOR were also improved. However, it is important to note that although evidence suggests that these are the central structures of these networks, clearly other brain regions are part of these networks including areas important for sensory, motor, motivational, attentional, and executive functions [50, 54].

### Effect of long-term voluntary exercise on cholinergic function of APP^NL-G-F^ mice

We performed brain immunostaining for ChAT^+^ cells to study cholinergic function. One-way ANOVA found a statistically significant difference in the number of ChAT^+^ cells (*F*_(2,10)_ = 9.049, *p* = 0.006, *η*^2^ = 0.644) among experimental groups. Mice in the APP-NE group showed a significant (*p* < 0.01) decrease in ChAT^+^ cells in the MSDB complex when compared with control mice (Fig. 9I). Figure 9I also shows that exercise intervention prevented the reduction in ChAT^+^ cells in the MSDB complex in comparison with APP-NE mice (*p* < 0.05) as indicated by Bonferroni post hoc analysis.

Consistent with our previous work [37], APP^NL-G-F^ mouse model of AD shows drastic reductions in ChAT^+^ cells in the MSDB. Importantly, the effects of physical exercise on the ascending cholinergic system that innervates the HPC were clear. The resulting enhanced cholinergic tone in HPC might be responsible for some of the functional recovery found following exercise intervention, like improvements in spatial learning and memory functions.

## Discussion

Regular physical exercise is associated with sustained cognitive functions as people age and may slow down or prevent the progression of various neurodegenerative diseases including AD [55]. In the present experiments, we tested the effects of long-term voluntary exercise for 9 months in a new generation knock-in mouse model of AD, the APP^NL-G-F^ model [28]. At the age of 12 months, the effects of this lifestyle treatment on learning and memory function and brain pathology associated with AD were evaluated. APP^NL-G-F^ mice allowed to voluntarily exercise showed an improvement in cognitive functions associated with various learning and memory networks [50, 56]. Brain pathology associated with AD was also impacted by long-term exercise including significant reductions in amyloid load, microgliosis, and preservation of ChAT^+^ cells (cholinergic function) in the brain of APP^NL-G-F^ mice. These profound reductions in brain pathology associated with AD are likely responsible for the observed improvement of learning and memory functions following extensive and regular exercise. These findings suggest potential of voluntary physical exercise to mitigate the cognitive deficits found in humans suffering from AD.

### Use of an AD mouse model that might more closely replicate the brain pathology found in human AD

Our lab has characterized the APP^NL-G-F^ mouse in several experiments and found that these mice show significant Aβ plaque through regions of neocortex (NC) and HPC, and indicate cognitive impairments at 6 months, but not earlier [37]. We showed that learning and memory functions associated with HPC, PRhC, and AMYG were compromised. The APP^NL-G-F^ mice also showed increased astrocytosis in the HPC, NC, MSDB, and other brain areas. Other brain changes in APP^NL-G-F^ mice included cholinergic and norepinephrine dysfunction [37]. These brain and behavioral changes are consistent with changes found in human AD patients supporting the use of APP^NL-G-F^ mice as an important improvement in available AD mouse models.

In the present study, we demonstrated the efficacy of long-term physical activity in reducing AD brain pathology and associated cognitive impairments in the APP^NL-G-F^ mice, a mouse model of AD that probably produces results that are more translatable to humans because of its similarity to human AD pathology.

### Effects of long-term voluntary exercise on various neural networks implicated in learning and memory functions

The APP^NL-G-F^ mouse model shows significant learning and memory impairments, and the pattern of impairments suggests that several memory networks are compromised [37]. These impairments include spatial learning and memory abilities dependent on a memory network centered on the HPC; novel object recognition dependent on a memory network centered on the PRhC [57, 58]; and fear conditioning processes dependent on a memory network centered on the AMYG [59–61]. We also showed that these same central brain regions of these networks exhibit many of the pathologies of human AD including Aβ pathology, microglial activation, and cholinergic dysfunction.

In the present experiment, we used this knowledge base about the mouse model of AD and evaluated the impacts of long-term voluntary exercise. This study was inspired by epidemiological and clinical studies showing that lifestyle changes like physical activity are a viable preventative approach for this form of age-related cognitive decline.

The potential of exercise to preserve cognitive functions in aging and age-associated brain diseases including dementia and AD has been reported in previous studies [14, 18, 20, 21]. These clinical findings were supported by several preclinical studies showing that physical activity improved the learning and memory functions in experimental models of AD [62–65].

The findings from the present study are consistent with other work assessing the benefits of exercise in mouse models of AD. One study reported that voluntary exercise for 16 weeks improved the memory function of Tg2576 mice and evidence also suggests that voluntary exercise over forced exercise was more effective [27] (using treadmill). In another study, results showed the beneficial effect of exercise in the Tg4-42 mouse model of AD on MWT and NOR tests, although it should be noted that these experiments combined exercise with an enriched environment [26]. Similarly, Adlard and colleagues also reported that exercise improved the learning and memory of TgCRND8 mice in the MWT [63]. The results from the present experiments combined with previous research suggest that long-term voluntary exercise reverses learning and memory functions dependent on neural networks centered on the HPC and PRhC. We also showed that long-term voluntary exercise improved fear conditioning to context in the APP^NL-G-F^ mice, although this improvement did not extend to cued fear conditioning. These results suggest that long-term voluntary exercise reverses, at least partially, the learning and memory network functions dependent on a neural network centered on the amygdala.

### Effects of long-term voluntary exercise on brain pathology associated with AD

In the present experiment, we found decreases in Aβ pathology in various brain regions such as MSDB complex, HPC, RSC, PRhC, and CCA of 12 months old APP^NL-G-F^ mice following long-term voluntary exercise. These brain regions have been implicated in different types of learning and memory functions [56, 57, 66–68]. The reduction in the amyloid burden in these areas may be responsible for improvement in the various behavioral tasks.

Other work using different mouse models of AD show similar effects [62, 64, 65, 69–73]. For example, physical exercise decreases levels of Aβ_40_ and Aβ_42_ in the HPC and AMYG of APP/PS1 transgenic mice [71]. In another experiment, Adlard and colleagues also reported that TgCRND8 mice with wheel running access for 5 months showed reduction in amyloid pathology which they argued might be responsible for the cognitive improvements observed [63]. Other studies are also consistent with this claim that exercise can reduce amyloid load [22, 27, 63].

Glial cell dysfunction observed in the postmortem human AD brain has been documented in various clinical studies [74, 75]. Additionally, several preclinical studies reported that physical exercise decreased astrocytes and microglial activation in experimental models of AD [70, 72, 73, 75]. Interestingly, in the present study, the long-term voluntary exercise manipulation only reduced microgliosis in specific brain regions like the HPC, PRhC, and MSDB. The latter effect is of specific interest as the MSDB complex provides cholinergic inputs to HPC and progressive deterioration of these projections are found in aging and neurodegenerative diseases including AD [32, 76–78], and we found the same effect in APP^NL-G-F^ mice [37]. In the present study, long-term voluntary exercise prevented the loss of ChAT^+^ cells in MSDB complex in 12-month-old APP^NL-G-F^ mice. A previous study also reported the beneficial effect of exercise on ChAT^+^ neurons in the MSDB of THY-Tau22 mice, a model of AD [69]. These findings indicate that long-term voluntary exercise aids in reducing inflammation in the AD brain. In the present study, the pattern of results suggests that the HPC, PRhC, and MSDB benefit the most from regular physical activity.

### APP mice, anxiety, and exercise

There is a significant amount of disagreement concerning the APP^NL-G-F^ mouse and whether they show an anxiety phenotype. Some researchers report that this AD mouse model does not show anxiety [79–82] and others do report an anxiety phenotype on some but not other measures [83, 84]. This is potentially relevant to the present study as it has been shown that exercise can reduce anxiety in humans although it is controversial in rodents [85]. If the APP^NL-G-F^ mice are anxious it is possible that the exercise treatment is reducing anxiety which could improve learning and memory performance separate from potential effects on plasticity and learning and memory function. For example, anxious mice might show elevated levels of thigmotaxia (not venturing away from the pool wall) which can impair MWT performance [45, 86]. If the exercise treatment reduces anxiety, this could be a reason for the improvements on the MWT. To assess this idea, we analyzed thigmotaxia during MWT training and found evidence that the APP^NL-G-F^ mice that were not given the exercise manipulation showed elevated thigmotaxic behavior compared to the controls or the APP^NL-G-F^ that were given the exercise treatment.

As noted above, thigmotaxia can emerge for a variety of reasons including, but not limited to, impaired learning and memory ability, anxiety, sex hormones, and executive functions. It is unclear in the present study, as in all other studies of this nature, what is driving the increase in thigmotaxia in the APP mice although impairments in learning ability and increased anxiety seem to be the most likely. One idea is that the positive effects of exercise on spatial learning and memory might be on neural networks important for controlling anxiety that are separate from the central structures of neural networks important for learning and memory functions examined in the present study. Another hypothesis is that impaired learning and memory functions render the subjects less confident in finding an escape route and they stay near the pool wall waiting to be removed by the experimenter [44]. This view emphasizes that the relationship between thigmotaxia and learning is not unidirectional. That is, elevated thigmotaxia can impair learning in the MWT but impaired learning can also increase thigmotaxia. A final hypothesis, one that we favor, is that the neural networks assessed in the present study that are implicated in learning and memory functions are also implicated in controlling fear responses and general anxiety [87–89] particularly ventral hippocampus, amygdala, and various parts of the prefrontal cortex. These systems are thought to control fear and anxiety by constraining fear responses to predictive contexts/cues predictive of aversive events, in other words via learning and memory functions [50]. According to this view, it is likely that the impacts of exercise on anxiety are via reduced pathology and dysfunction of the hippocampus, amygdala, and prefrontal cortex and associated cognitive functions. This analysis is consistent with the claim that voluntary exercise improved learning and memory functions at least partially via reductions in anxiety and reduced various brain pathologies associated with AD in medial temporal lobe brain regions thought to be central in complex neural networks supporting various forms of memory that control fear and anxiety responses.

Further research focused on disentangling the different hypotheses about the relationship between memory impairments associated with AD, anxiety, and treatment effects is required to fully understand this complex issue. This work would include an assessment of the effects of treatments in AD models, on dorsal versus ventral hippocampal pathology, and on their relationship to improved memory and/or reduced anxiety.

### Strengths of the current approach

The current experiment is unique and may represent an advancement in our understanding of the efficacy of long-term exercise in preventing the rapid descent into dementia in AD. First, the current approach used a new generation mouse model of AD. Second, this study focused on long-term physical activity in isolation versus in combination with other strategies like environmental enrichment and cognitive training. Third, we employed a battery of learning and memory tasks as functional assays for different learning and memory networks. Finally, we assessed a wider panel of pathologies associated with AD in these brain networks.

### Limitation of the study

In the present study, we have provided strong evidence for the therapeutic potential of non-pharmacology strategies for the management of AD. Although, we have revealed promising results showing protective effect of long-term exercise against cognitive impairment and pathology of AD, still, there are few limitations to the present study. First, negative littermates of APP^NL-G-F^ mice have not been used as a normal control. We used C57BL/6 as a normal control as APP^NL-G-F^ mice are generated on a C57BL/6 background. Looking at the research literature using this AD mouse model, a lot of researchers use C57’s as controls for APP^NL-G-F^ mice. However, if you look at the paper by Nilsson, Saito, and Saido (2014), the authors suggest that because APP^NL^ mice exhibit the same levels of CTF-beta as APP^NL-F^ and APP^NL-G-F^ mice, they are the proper negative controls [90]. In our view, the use of the APP^NL^ mice as the negative controls might not be appropriate as they have one of the mutations associated with AD pathology. For the present study, these negative controls were not available in our lab at the time. We currently have these mice in house now and will use them in future experiments with these caveats in mind. Still, we found that non-pharmacological (long-term exercise) manipulation reduced brain pathology and improved cognitive functions of this knock-in mouse model of AD. Second, normal control and APP-NE groups were not provided with a static wheel in same cage for 9 months. Ideally, this experimental group should be included in future studies to rule out the potential additive effect of environmental enrichment due to the presence of the wheel in the APP-Ex group. We predict that this type of experimental design would replicate the present results showing the beneficial effect of exercise only in the APP-Ex group and no contribution of the presence of an inoperable running wheel in the home cage. Additionally, we did not record the extent of exercise in the present study, so we could not correlate the level of exercise of an individual with the behavioral parameters and pathological makers studied in the present study. Third, another potential caveat associated with the design of the present study is that the subjects that were given the exercise treatment were socially isolated during this time. It is possible that social isolation might have contributed to the reductions in brain pathology [91]. However, we think that this is unlikely as our reading of the literature suggests that social isolation should impair hippocampal plasticity and learning and memory function [92–94]. Our view is that these effects would have worked against the beneficial effects of exercise and yet we still found profound impacts of the exercise manipulation on brain pathology, anxiety, and learning and memory functions. Finally, we selected 4–5 mice from each group for histopathological assessment. This means that not all the brains from subjects included in the behavioral analysis were assessed. We have consistently (37) found that this number of subjects for pathological assessments is large enough and the patterns of effects, in the AD mouse model we use, over many studies (onset of pathology, maximum threshold levels, location). However, it is important for the reader to note this when interpreting the data, as well as that this design made it difficult to correlate long-term exercise-induced improvement in behavioral outcomes and brain pathology.

Future work should consider these limitations when designing future experiments assessing the impacts of exercise on AD pathology and associated cognitive impairments.

## Summary

The present experiments provide strong causal evidence that long-term voluntary exercise significantly reverses severe cognitive impairments, anxiety, and associated pathological changes in the brain of a new generation knock-in model of AD. Several possible mechanisms involved in protective effect of exercise against AD include reduction in oxidative stress [95, 96], neuroinflammation [70, 72, 73, 75], amyloid pathology [22, 27, 63, 71], and improvement in cerebral blood flow [97, 98]. Physical exercise enhanced neurogenesis, synaptogenesis, and cholinergic cell’s function may be responsible for improvement in various cognitive functions including learning and memory [64, 69, 97–100]. Although, out of these several mechanisms, we have only studied the neuroinflammation, amyloid pathology, and cholinergic cells integrity, however, involvement of other mechanisms in the protective effect of long-term exercise cannot be ruled out. The present results combined with our analysis of the existing research literature suggests that implementing exercise in combination with other lifestyle factors like cognitive training or diet could be effective non-pharmacological approaches to prevent or delay the progression of AD. Future work will be directed at assessing the effects of other lifestyle preventative measures alone or in combination with voluntary exercise.

## Data Availability

The datasets used and/or analyzed during the current study are available from the corresponding author on reasonable request.

## Abbreviations

Aβ: Amyloid-beta
AD: Alzheimer’s disease
AMYG: Amygdala
APP: Amyloid precursor protein
APP-KI: APP knock-in
CAA: Cortical amygdalar area
ChAT: Choline acetyl transferase
CTF-β: C-terminal fragment-β
FC: Fear conditioning
HPC: Hippocampus
ITI: Intertrial interval
MSDB: Medial septum-diagonal band complex
mPFC: Medial prefrontal cortex
MWT: Morris water task
NFT: Neurofibrillary tangles
NOR: Novel object recognition
PBS: Phosphate-buffered saline
PFA: Paraformaldehyde
PRhC: Perirhinal cortex
PS1: Presenilin1
RSA: Retrosplenial area
TBS: Tris-buffered saline
